# Refractory malignant glaucoma after secondary IOL implantation in an eye with 17-year-old vitrectomy: A case report

**DOI:** 10.1097/MD.0000000000046914

**Published:** 2026-01-09

**Authors:** Zhonghua Sun, Zhen Ji, Cuijuan Liu, Miaomiao Zhang, Chenming Zhang, Shanshan Ren, Lei Gao, Wei Liu

**Affiliations:** a Department of Ophthalmology, Jinan Second People’s Hospital, Jinan, China; b Nova Southeastern University College of Optometry, Miami, FL.

**Keywords:** ciliary body-zonules-crystalline lens-hyaloid-anterior vitreous complex, irido-zonulo-hyaloid vitrectomy, malignant glaucoma, post-vitrectomy recovery, pseudophakia

## Abstract

**Rationale:**

Malignant glaucoma (MG) results from aqueous misdirection and entrapment in the vitreous cavity. This process drives anterior displacement of the lens-iris diaphragm, causing uniform shallowing of the central and peripheral anterior chamber, typically with elevated intraocular pressure (IOP). Clear lens extraction with anterior vitrectomy remains the definitive intervention.

**Patient concerns:**

We report a rare case of MG following combined pars plana vitrectomy (PPV) and intraocular lens (IOL) implantation and detail its management. A 49-year-old Chinese male presented with a history of ocular trauma in the left eye, which was treated 17 years ago by PPV with lensectomy. After secondary IOL implantation with pupilloplasty was successfully performed, the patient experienced recurrent episodes of shallow anterior chamber and elevated IOP (peaking at 44 mm Hg), which could not be resolved by maximal medical therapy, suture-leakage assisted incision, or laser peripheral iridotomy.

**Diagnoses:**

The condition was considered postoperative MG.

**Interventions:**

The patient underwent a second surgery, which was trabeculectomy combined with partial irido-zonulo-hyaloid-vitrectomy.

**Outcomes:**

Subsequent follow-up demonstrated stable anterior chamber depth and normalized IOP.

**Lessons:**

To resolve MG, the fundamental objective must be restoring pressure equilibrium across the ciliary body-zonules-lens-hyaloid-anterior vitreous complex. Achieving this requires reconstructing the complex’s structural integrity and normalizing its regulatory function in aqueous humor circulation.

## 1. Introduction

Malignant glaucoma (MG) is a vision-threatening surgical complication characterized by aqueous misdirection into the vitreous cavity. Contemporary data report 2% to 4% incidence after angle-closure glaucoma surgery.^[1]^ The high-risk anatomical features include short axial length, shallow anterior chamber, small cornea, large lens, and narrow anterior chamber angle. These features create a “crowded anterior segment” that potentiates post-surgical aqueous misdirection.^[2]^ The pathophysiology of MG involves dysfunctional vitreociliary pressure gradients, which permit anterior hyaloid vaulting and consequently cause the trapping of aqueous humor within the vitreous cavity. This entrapment forces anterior lens-iris displacement, causing generalized shallowing of central and peripheral anterior chambers with or without elevated intraocular pressure (IOP).^[3]^ As a result, patients with MG are at risk of sight-threatening complications (e.g., corneal decompensation, optic atrophy, and permanent blindness^[4,5]^), and prompt surgical interventions such as clear lens extraction combined with anterior vitrectomy are needed.^[6,7]^

This paper reports a rare case of MG occurring after secondary IOL implantation in a patient with favorable anterior chamber and iris conditions. We attribute the occurrence and development of MG to the partial deficiency or disruption caused by trauma, as well as the further choroidal edema, the complex inflammation of CZLHV, and the ciliary body congestion caused by the recent IOL fixation. Our therapeutic success underscores the physiological significance of the ciliary body-zonules-crystalline lens-hyaloid-anterior vitreous complex (CZLHV), a structural and functional interface between the posterior chamber and the vitreous cavity that plays a crucial role in IOP dynamics and fluid compartmentalization within the eye, in MG pathogenesis.

## 2. Case description

A 49-year-old man sought evaluation for persistent blurred vision in the left eye. Seventeen years prior, the patient suffered an intraocular foreign body injury, which required the surgical removal of the foreign body, pars plana vitrectomy, and lensectomy. His vision failed to improve postoperatively. He reported no significant medical history, no use of systemic medications, and no additional surgeries. He specifically denied any history of glaucoma or other ocular pathology.

**Figure 1.**
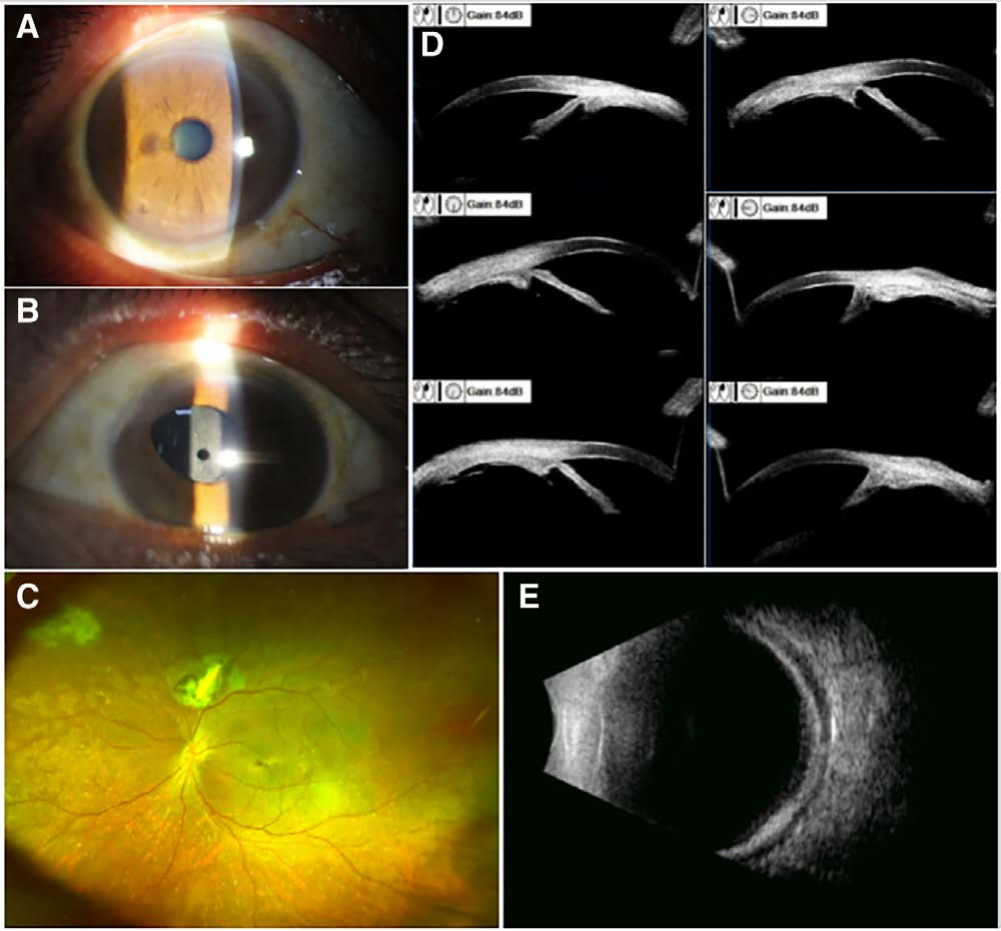
Preoperative examinations. (A) Right eye: Moderately deep anterior chamber, round pupil (3 mm diameter), transparent lens. (B) Left eye: Clear cornea, deep anterior chamber with transparent aqueous. Partial iris atrophy (9–11 o’clock) with anterior synechiae; irregular pupil (~4 mm diameter, superonasally displaced). Crystalline lens absent; intact capsular bag with mild opacification and central clear aperture. (C) Fundus: Flat, attached retina; well-defined optic disc with normal coloration. Superior chorioretinal scar with old laser spots above optic disc. (D) UBM: Normal ciliary body morphology, iridocorneal adhesion (9–11 o’clock); open angle structures elsewhere; a small amount of vitreous tissue visible on the flat portion. (E) B-scan: Choroidal edema with shallow detachment.

The following exams were completed upon admission. Best-corrected visual acuity (BCVA) was 65/80 (OD) and counting fingers at 10 cm (OS). Non-contact tonometry showed intraocular pressure (IOP) of 11 mm Hg in both eyes. Slit-lamp examination of the right eye revealed a moderately deep anterior chamber with well-defined iris texture, a round pupil (3 mm diameter) reactive to light, a transparent lens, and unremarkable fundus findings (Fig. 1A). For the left eye, slit-lamp examination demonstrated a clear cornea with deep anterior chamber and clear aqueous humor. It also identified partial iris atrophy with anterior iris synechiae from 9 to 11 o’clock, an irregular superonasally displaced pupil (~4 mm diameter), and aphakia with an intact capsular bag exhibiting a mild opacification and a central clear aperture (Fig. 1B).

Additional examinations were performed on the left eye. Fundus examination revealed a flat, well-attached retina and an optic disc with distinct margins and normal color, and superior scar tissue encircled by laser spots was noted (Fig. 1C). Axial length measurement using IOLMaster 700 (Carl Zeiss AG, Germany) yielded 25.55 mm. Ultrasound biomicroscopy (UBM; SW3200L, Tianjin Suowei Electronic Technology Co., Ltd., China) revealed anterior iris adhesion to the corneal endothelium at 9 to 11 o’clock, with open angles in the other quadrants; the ciliary body morphology appeared normal, and a small amount of vitreous tissue was visible on the flat portion (Fig. 1D). Quantitative ultrasonography (Echograph Aviso, Quantel Medical, Cournon-d’Auvergne, France) detected choroidal edema with shallow detachment (Fig. 1E). Specular microscopy (SP-3000P, Topcon, Tokyo, Japan) documented corneal endothelial density at 2433 cells/mm^2^.

After preoperative evaluations, the patient underwent secondary intraocular lens (IOL) sulcus fixation combined with pupilloplasty in the left eye. An experienced ophthalmologist implanted a Softec HDO IOL (Lenstec (Barbados) Inc., Christ Church, Barbados) into the ciliary sulcus. The moderately dilated and fixed pupil from previous trauma was addressed with a single 10-0 polypropylene suture (Ethicon W2790; Johnson & Johnson, Raritan, NJ, USA) for iris fixation to reduce pupillary diameter. Intraoperatively, significant residual anterior vitreous and posterior capsular opacification were observed. Consequently, central anterior vitrectomy and posterior capsulotomy were performed to optimize postoperative visual quality. Triamcinolone acetonide was used to confirm absence of residual vitreous in the IOL area and the pupillary aperture.

**Figure 2.**
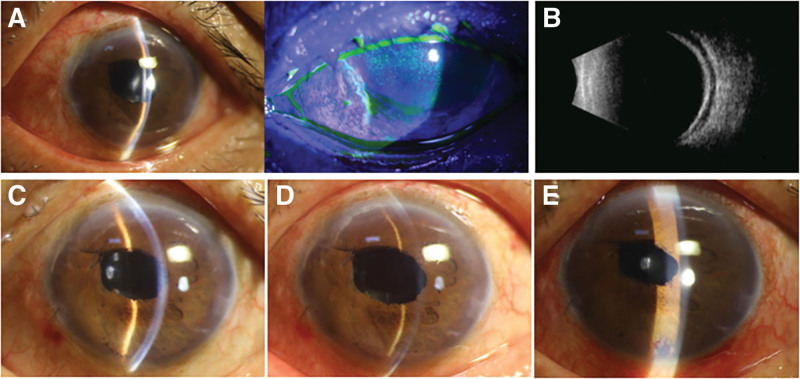
Initial postoperative examinations. (A) Postoperative Day 1, slit lamp: Conjunctival congestion, corneal edema, grade II shallow AC; iris suture (11 o’clock); irregular pupil (~3 mm); well-positioned IOL; clear central posterior capsule. Fluorescein: Seidel positive (9 o’clock side port). (B) Day 1, B-scan: Choroidal edema with shallow serous detachment. (C) Day 2, slit lamp: Satisfactory AC depth. (D) Day 3, slit lamp: Fluctuating recurrent AC shallowing. (E) Day 4, post-LPI exam: Patent inferior iridotomy, deep AC.

The postoperative findings for the left eye were as follows. On Day 1, the best-corrected visual acuity (BCVA) was 20/200. Conjunctival congestion, corneal edema, and a grade II shallow anterior chamber with a suture were visible at the 11 o’clock iris position. The pupil exhibited irregular morphology with an approximate diameter of 3 mm. The IOL was well-positioned, and the central opening of the posterior capsule remained clear. The IOP was 33 mm Hg. A positive Seidel sign was detected on fluorescein staining at the 9 o’clock side port (Fig. 2A). However, despite satisfactory closure of the 9 o’clock side port incision, the anterior chamber continued to have difficulty forming and IOP fluctuated between 40 and 44 mm Hg. A comprehensive regimen was then initiated, including topical antiglaucoma agents (brinzolamide, timolol, brimonidine tartrate) and systemic mannitol injection. Additionally, intravenous systemic glucocorticoids were administered for anti-inflammatory treatment. Subconjunctival mydriatics (atropine and epinephrine hydrochloride) were employed to facilitate anterior chamber reconstruction. Subsequent evaluations showed improved anterior chamber depth relative to baseline, with IOP stabilizing at 7 to 16 mm Hg. However, ocular B-scan ultrasonography documented persistent choroidal edema and shallow detachment (Fig. 2B).

On Day 2, slit-lamp examination revealed a well-maintained anterior chamber depth (Fig. 2C) with an IOP of 15.5 mm Hg. Close monitoring was continued, and on Day 3, there was recurrent anterior chamber shallowing with depth fluctuations (Fig. 2D). Therefore, laser peripheral iridotomy (LPI) was performed on Day 4 to establish anterior-posterior chamber communication. The procedure improved the anterior chamber stability (Fig. 2E) and maintained the IOP within normal limits. At discharge (Day 6), the left eye exhibited a mildly shallow anterior chamber; BCVA was 20/200 and IOP was 16 mm Hg. The patient continued topical pranoprofen, prednisolone acetate, compound tropicamide, and tobramycin eye drops.

**Figure 3.**
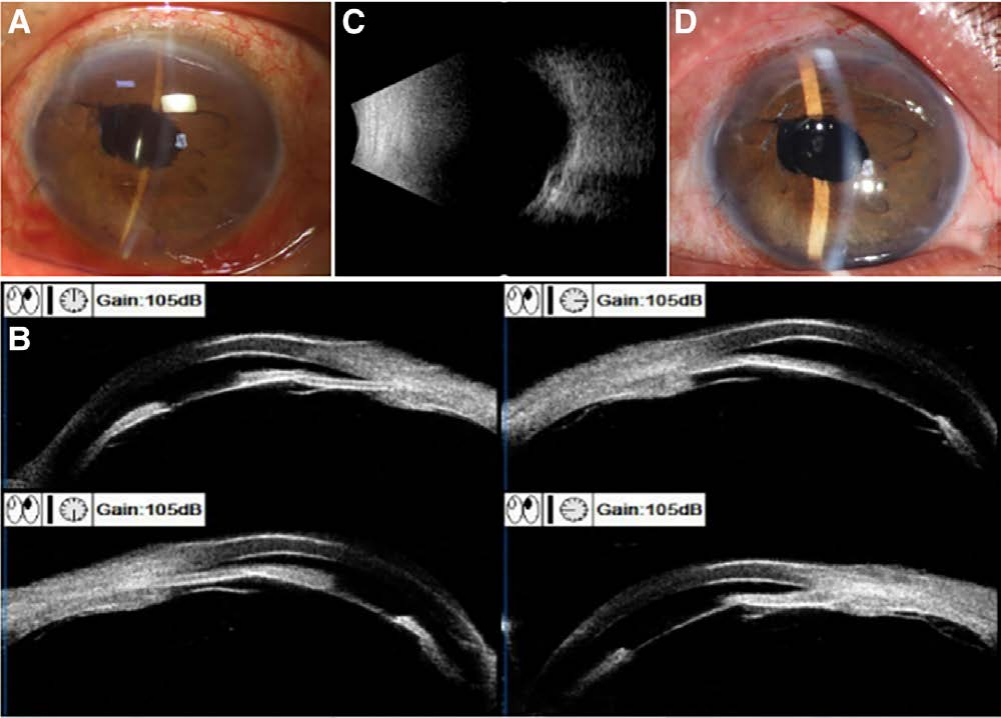
Examinations related to re-admission. (A) Re-admission, slit lamp: Conjunctival congestion; clear cornea; corneal suture (7 o’clock); shallow AC; partial iris atrophy; patent LPI (11 o’clock); irregular pupil (~3 mm, nasosuperior displacement); IOL adherent to iris; clear central capsulotomy. (B) Re-admission, UBM: Ciliary forward displacement; complete IOL-iris contact; anterior iris-IOL diaphragm displacement; extremely shallow AC (peripheral obliteration); angle closure. A small amount of vitreous tissue is visible on the flat portion. (C) Post-revision, B-scan: Resolved choroidal edema. (D) 3-month follow-up, slit lamp: Clear cornea; moderate AC depth; patent superior LPI; well-positioned transparent IOL.

The patient presented to the outpatient clinic 20 days after the discharge. The chief complaints were left ocular distension, visual acuity decline, and headache persisting for 1 week. The right eye had a BCVA of 20/80 and an IOP of 11 mm Hg, but the left eye had a visual acuity of counting fingers at 10 cm and an IOP of 21 mm Hg. Slit-lamp examination of the left eye (Fig. 3A) demonstrated conjunctival congestion and a transparent cornea with a suture at the 7 o’clock position. The anterior chamber was shallow, with partial iris atrophy and an intact suture at the 11 o’clock position. A patent LPI incision was observed inferiorly. The pupil was irregular (3 mm diameter) and deviated superonasally. Iris-IOL adherence was noted, the central capsulorhexis opening remained transparent, and the vitreous cavity was clear. Fundus examination findings were unchanged from previous records. The endothelial cell count was 2215 cells/mm^2^. The following were detected by UBM: ciliary body forward displacement, iris-IOL contact with anterior displacement of the iris-lens diaphragm, an extremely shallow anterior chamber (complete disappearance peripherally), and complete angle closure. A small amount of vitreous tissue was visible on the flat portion (Fig. 3B).

The diagnosis of MG was confirmed, necessitating readmission for surgical intervention. Given the 360° angle closure, the patient underwent irido-zonulo-hyaloid-vitrectomy (IZHV) combined with trabeculectomy. The procedure included peripheral iridectomy, partial zonulectomy, anterior hyaloid incision, and anterior vitrectomy. The surgical procedure was as follows. A curved conjunctival incision was made at the 12 o’clock limbus, and the fascial tissue toward the fornix was then dissected. A 3 × 4 mm lamellar scleral flap was created at 12 o’clock, extending 1 mm into clear cornea. Sponges soaked in mitomycin C (MMC, 0.4 mg/mL) were applied under conjunctival and scleral flaps for 3 minutes, followed by thorough irrigation with balanced saline solution. An anterior chamber paracentesis was created at 3 o’clock to facilitate aqueous drainage. A vertical limbal incision was made beneath the scleral flap, and a 1 × 2 mm corneoscleral tissue segment was excised through this incision. A peripheral iridectomy was then performed adjacent to the excision site. Afterwards, the vitrectomy probe was introduced through the iridectomy to excise suspensory ligaments and surrounding tissues in a superior-to-inferior direction. Immediate fluid egress reformed the anterior chamber, repositioning the iris and IOL. Three lamellar scleral sutures (one adjustable) were placed, and the bulbar conjunctiva was closed with interrupted sutures.

The postoperative course was unremarkable, with a maintained anterior chamber depth and stable IOL positioning. Serial B-scan ultrasonography demonstrated progressive resolution of choroidal edema (Fig. 3C). The patient was discharged on postoperative Day 4 with an IOP of 17 mm Hg. At 3-month follow-up, BCVA in the left eye was 20/30. Ocular examination revealed mild conjunctival injection, transparent cornea, moderately deep anterior chamber, patent superior peripheral iridotomy, and stable IOL position (Fig. 3D). The IOP stabilized at 15 mm Hg, and no additional therapy was required.

## 3. Discussion

Because of its unique pathophysiology involving the misdirection of aqueous humor, MG responds poorly to standard antiglaucoma medications. Its primary manifestations include elevated IOP, severe shallowing of peripheral and central anterior chambers, and anterior displacement of the iridolenticular diaphragm. Current evidence attributes MG to retrograde aqueous flow trapped within the vitreous cavity,^[8]^ and the postoperative onset of MG is common.^[9]^ Theories such as lens block,^[10]^ ciliary block,^[11]^ and vitreous block tackle MG based on isolated mechanisms.^[12]^ Consequently, conventional surgical interventions adopt iridotomy to address pupillary block,^[13]^ lens extraction to relieve lens block,^[14]^ posterior capsulotomy/pars plana vitrectomy to mitigate vitreous block,^[15]^ and transscleral cyclophotocoagulation to reduce aqueous production in ciliary block.^[16]^ Although these surgical approaches have demonstrated therapeutic effects to varying degrees, their efficacy remains inconsistent.^[17]^ Phacoemulsification with anterior vitrectomy has emerged as a predominant modality,^[18]^ but in eyes with anteriorly positioned ciliary bodies or abnormal ciliary body-lens proximity, anterior vitrectomy fails to overcome anterior hyaloid membrane resistance and yields a success rate of only 64%.^[19]^ Physicians now realize that anterior lens displacement is not the direct cause of MG but a secondary phenomenon, and pupillary/ciliary/vitreous blocks are consequences rather than initiating factors. Indeed, the present case strongly challenges conventional theories. Although the patient previously had trauma-induced lens extraction, anterior vitrectomy, and residual vitreous excision before IOL implantation, he still developed refractory shallow anterior chamber and elevated IOP. The progression of his condition further underscores the limitations of current pathophysiological theories, suggesting that interventions directly targeting vitreociliary pressure gradients are needed.

Currently, MG is being reframed as “aqueous misdirection syndrome,” i.e., a unified pathophysiology involving dysfunctional pressure gradients across the vitreociliary interface. High-resolution UBM and anterior segment optical coherence tomography (AS-OCT) validate the new model, revealing anterior hyaloid vaulting and ciliary body edema as pathognomonic features. Applying the aqueous misdirection framework to surgical design improves therapeutic outcomes. The CZLHV complex, which integrates the iris, lens, ciliary body, vitreous, and choroid, orchestrates intercompartmental fluid dynamics and is central to MG progression. The IZHV procedure disrupts the CZLHV complex to directly reset vitreociliary pressure gradients and has proven highly effective.^[20]^ The present case further illustrates that the CZLHV complex is crucial in the pathogenesis of MG. When a classic surgical approach consisting of phacoemulsification, IOL implantation, central capsulorhexis, and anterior vitrectomy yields suboptimal results, adjunctive peripheral zonulectomy and peripheral capsulotomy (both of which are core IZHV components) should be employed to enhance success.

Our patient developed MG following secondary IOL sulcus fixation, and we hypothesize the pathological progression as follows. The trauma 17 years ago may have caused partial zonular deficiency or disruption; indeed, a small, stable posterior capsular rent was observed. Although the origin of this rent could not be definitively determined, we consider 2 likely causes. The removal of an intraocular foreign body through the pupil or via the flat part of the ciliary body could have abraded or punctured the posterior capsule.^[21]^ Alternatively, an iatrogenic tear of the posterior capsule may have occurred during the prior vitrectomy or lensectomy if the vitrector contacted the capsule or if the irrigation/aspiration tip engaged the capsular margin.^[22,23]^ Upon admission, the patient presented with choroidal edema, which is thought to relate to blood-retinal barrier disruption from inflammation and to metabolic changes in the retina and choroid after prolonged light exposure in the absence of a crystalline lens.^[21]^ The recent IOL fixation and pupilloplasty further stimulate the iris-choroid tissue, acting as a surgical trigger that induces acute inflammation. This resulted in further choroidal expansion and exacerbated congestion and edema of the ciliary body. Consequently, inflammation exudates enveloped the CZLHV complex and obstructed fluid drainage from the vitreous cavity to the anterior chamber. The unopposed aqueous production elevates the posterior segment pressure, causing anterior displacement of the CZLHV complex. Meanwhile, the concurrent choroidal edema with shallow detachment establishes a sustained high-pressure environment, facilitating fluid transmission within the vitreous cavity.^[2]^ The residual vitreous can obstruct outflow after an inadequate anterior vitrectomy,^[17]^ and the obstruction triggers progressive lens-iris displacement, causing anterior chamber shallowing. Subsequently, the IOL-iris adherence blocks posterior-to-anterior aqueous flow. Ultimately, continuous aqueous production forms a vitreous aqueous pocket, which is the hallmark of MG. Therefore, for patients at high risk of MG, proactive perioperative anti-inflammatory therapy is clinically imperative. For example, aggressive systemic and topical corticosteroids can be initiated preoperatively and continued postoperatively to suppress inflammation. Studies confirm that proactive medical management prevents full MG development in approximately 50% of high-risk cases.^[3]^

In the present case, the patient experienced preexisting choroidal edema before the surgery. The rapid execution of the procedure before the edema had completely resolved, combined with the concurrent performance of iridoplasty during IOL implantation, may have introduced potential risks and surgical complications that could lead to secondary MG. We drew the following practical lessons from this case. In clinical practice, patients with choroidal edema using fundus angiography or other targeted studies to determine the etiology and, when indicated, initiate preoperative anti-inflammatory therapy. We defer elective intraocular procedures until significant choroidal edema has resolved and avoid performing concurrent iridoplasty during IOL implantation unless absolutely necessary.

Delayed or absent surgical treatment of MG worsens intraocular inflammation and leads to a poorer outcome. Thus, when medical therapy fails, prompt surgical intervention is indicated. The IZHV procedure effectively establishes anterior chamber-vitreous cavity communication and is currently the gold standard for MG treatment.^[3,24]^ In MG, the zonular apparatus is both a mediator of pathology and a target of therapy.^[18]^ In eyes with inadequate anterior chamber formation post-vitrectomy (via posterior capsular approach through the central lens region), 2 barriers must be disrupted to treat MG: the capsule-IOL complex within the capsular bag and the structural integrity of the vitreous zonular ligaments. This dual disruption establishes aqueous outflow between vitreous and posterior chambers.^[19]^ In this case of MG, IZHV restored anterior chamber anatomy while trabeculectomy created an external drainage channel for IOP control and corrected the 360° closure.^[25]^ The combined procedure (trabeculectomy and IZHV) successfully resolved the MG. However, a comprehensive analysis of this case revealed that the patient lacked gonioscopy to further evaluate the angle status. Surgical decision-making should always be grounded in comprehensive preoperative assessments, and in clinical practice, adhering to the “less is more” principle is essential. It is possible that preoperative gonioscopy would have shown findings amenable to simple goniosynechialysis, which might have relieved the recently formed angle closure and avoided more invasive surgery. These experiences hold significant implications for our future clinical work.

## Author contributions

**Conceptualization:** Zhonghua Sun, Chenming Zhang, Wei Liu.

**Data curation:** Zhonghua Sun, Zhen Ji, Cuijuan Liu, Miaomiao Zhang, Wei Liu.

**Formal analysis:** Zhonghua Sun, Zhen Ji, Cuijuan Liu, Lei Gao.

**Funding acquisition:** Miaomiao Zhang, Chenming Zhang, Wei Liu.

**Resources:** Miaomiao Zhang, Wei Liu.

**Software:** Cuijuan Liu, Shanshan Ren, Lei Gao.

**Validation:** Chenming Zhang.

**Writing – original draft:** Zhonghua Sun, Zhen Ji, Cuijuan Liu.

**Writing – review & editing:** Zhonghua Sun, Zhen Ji, Miaomiao Zhang, Shanshan Ren, Lei Gao, Wei Liu.
